# The role of PTP1B (PTPN1) in the prognosis of solid tumors: A meta-analysis

**DOI:** 10.1097/MD.0000000000030826

**Published:** 2022-10-07

**Authors:** Jiupeng Zhou, Hui Guo, Yongfeng Zhang, Heng Liu, Quanli Dou

**Affiliations:** a Xi’an Chest Hospital, Xi’an, Shaanxi Province, China; b The First Affiliated Hospital of Xi’an Jiaotong University, Xi’an, Shaanxi Province, China.

**Keywords:** meta-analysis, prognosis, PTP1B (PTPN1), tumor

## Abstract

Protein tyrosine phosphatase 1B (PTP1B) played different role in different solid tumors, and was associated with the prognosis of solid tumors. However, the roles existed controversy. This meta-analysis was performed to determine whether PTP1B was relevant to the prognosis of solid tumors.

**Materials and methods:**

A literature search in Web of Science, Embase and PubMed databases were performed up to November 1, 2021. A meta-analysis dealed with PTP1B assessment in solid tumors, providing clinical stages and survival comparisons according to the PTP1B status.

**Results:**

High PTP1B expression was significantly associated with later clinical stage of solid tumors (Odds ratio [OR] 2.25, 95% confidence interval [CI]: 1.71–2.98, *P* < .001). For solid tumors, the hazard ratio (HR) for disease free survival (DFS) detrimental with high PTP1B expression compared with low PTP1B expression was 1.07 (95%CI: 0.67–1.73, *P* = .77) with the obvious heterogeneity (*P* = .03, *I*^2^ = 66%). The HR of overall survival (OS) for solid tumors with high PTP1B expression versus low PTP1B expression was 1.26 (95%CI: 1.03–1.55, *P* = .03) with significant publication bias (*t* = 3.28, *P* = .005). Subgroup analysis indicated that the high expression of PTP1B was remarkably correlated with poor OS in colorectal carcinoma, only (HR = 1.43; 95%CI: 1.18–1.74; *P* = .003).

**Conclusions:**

High PTP1B expression is significantly associated with later clinical stage of solid tumors. The high expression of PTP1B is remarkably correlated with poor OS in colorectal carcinoma, only. There is no definite conclusion that PTP1B was, or not associated with DFS and OS of solid tumors because of heterogeneity and publication bias. Whether PTP1B can be used as a biomarker for predicting the prognosis of solid tumors needs further study.

## 1. Introduction

Protein tyrosine phosphatase 1B (PTP1B), also named as tyrosine protein phosphatase non receptor type 1, is the first discovered member of the protein tyrosine phosphatase family. It is encoded by the human PTPN1 gene.^[1,2]^ Recent reports show that PTP1B produces a marked effect on many diseases including diabetes mellitus,^[3]^ cancer,^[4,5]^ autoimmune diseases, neurodegenerative diseases^[6]^ and hepatic diseases.^[7]^

PTP1B plays an oncogenic role in non-small cell lung cancer, gastric cancer, prostate cancer, colorectal cancer, and liver cancer, and is associated with poor prognosis by up regulating oncogenes or down regulating tumor suppressor genes.^[8–12]^ It is also believed that PTP1B is not related to the poor prognosis of solid tumors.^[13]^ On the contrary, PTP1B down regulates breast tumor kinase and insulin-like growth factor 1 receptor signals as a negative regulator in ovarian cancer cells^[14]^ and is related to good prognosis.^[15,16]^ In addition, PTP1B plays an antitumor role in B-cell lymphoma.^[17]^ Therefore, the role of PTP1B in tumorigenesis may be decided by tumor type.^[18]^ However, it remains unclear whether this different effect of PTP1B on prognosis is caused by limited sample sizes or real difference. Herein we performed a meta-analysis of investigating the prognostic value of SHP-2 expression in solid tumor patients.

## 2. Materials and Methods

A literature search in Web of Science, Embase, and PubMed databases for published studies were performed up to November 1, 2021. Studies were selected using the keywords: “PTPN1,” “PTP1B,” “cancer,” “tumor,” “mortality,” “prognosis,” “survival,” and “outcome.” The bibliography of the article was also checked manually to avoid missing additional research. The study obtained approval from the Ethics Committee of Xi’an Chest Hospital.

### 2.1. Selection criteria

The inclusion criteria were as follows: PTP1B expression was examined by immunohistochemistry or real-time quantitative polymerase chain reaction; dichotomous model analyzed the association of PTP1B expression with solid tumors clinical stages or prognosis; prognostic correlation literatures had adequate data to evaluate the hazard ratio (HR) and 95% confidence interval (CI) between PTP1B expression and clinical outcomes. The exclusion criteria were as follows: Review articles, letters or experiments on animal models or human cell lines; Insufficient information to evaluate HR. When 2 or more publications reported on the same patient population, only the most integrated or recent studies were selected.

### 2.2. Data extraction and quality assessment

All eligible literatures were examined by 2 authors independently, and then the data were extracted. The following information was extracted using a specially designed form: the first author, year of publication, country, sample size, clinical stages, PTP1B assessment methods, cutoff value of PTP1B, follow-up time and survival data. HR was first extracted from multivariate analysis if feasible, otherwise, HR from univariate analysis. If this information was missing, HR was estimated from the Kaplan–Meier curve using the method reported by Tierney et al^[19]^ and Parmar et al.^[20]^ If there was a dispute between the 2 researchers, the third researcher should participate in the discussion until an agreement was reached. Newcastle Ottawa quality assessment scale was adopted for quality evaluation. A score of 6 or more was considered high quality.

### 2.3. Statistical analysis

All statistical analyses were performed by Stata 13.0 and Review Man 5.3. Data on PTP1B expression predicting overall survival (OS) and disease free survival (DFS)/progression free survival (PFS) were pooled across studies. The relationship between PTP1B expression and clinical stages was summarized by estimated odds ratio (OR). In the process of merging data, Q statistic tested the heterogeneity. The degree of heterogeneity was expressed by *I*^2^ value. *P* value ＜.10 and/or *I*^2^ ＞ 50% were considered significant heterogeneity, using random effect model. Otherwise, the fixed effect model was used. Publication bias was assessed by Egger test. *P* < .05 indicated significant difference.

## 3. Results

### 3.1. Studies identification and characteristics of eligible studies

**Figure 1.**
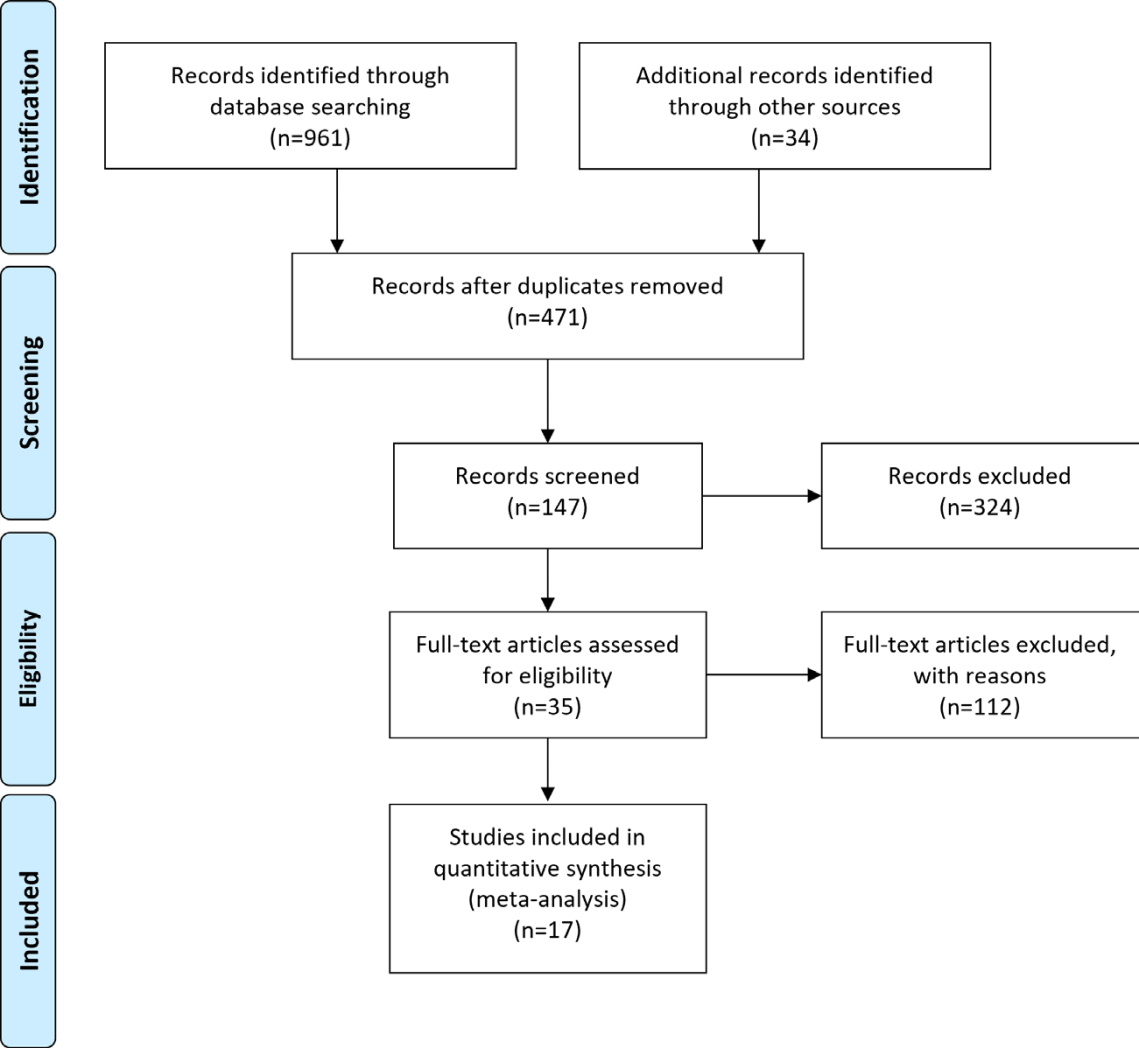
A flowchart describing the procedures of document retrieval and selection.

Totally 961 articles about PTP1B and solid tumors were found by database retrieval. However, 926 studies that were repeatedly reported, irrelevant to our purpose or without clinical samples were excluded. Of the remaining 35 studies, 18 were excluded after full-text evaluation those were reviews, case reports or did not provide sufficient data. Ultimately, 17 studies entered the final meta-analysis including a total number of 4188 patients (Fig. 1).

The average sample number of patients every study was 209.4 (range: 29–1402). They respectively came from Italy, China, Switzerland, Netherlands, Spain and Norway. In this meta-analysis, twelve different cancer types were contained, which respectively was 1 bladder cancer, 2 esophagus carcinoma, 3 breast cancer, 4 colorectal cancer, 2 gastric carcinoma, 1 neuroblastoma, 1 glioma, 1 pancreatic carcinoma, 2 non-small cell lung carcinoma, 1 serous carcinoma, 1 malignant mesothelioma and 1 malignant melanoma. Eight studies reported clinical stages, 4 studies estimated DFS, and 16 studies proposed OS (Table 1).

**Table 1:** The basic information and data of all included studies in the meta-analysis.

Author (yr)	Country	Cancer type	Total number	PTP1B expression		TNM stage		OS				DFS				Detection method	Criterion of high expression	Quality stars (NOS)
						I/II	III/IV	HR	95% CI	In (HR)	Se((InHR))	HR	95% CI	In(HR）	Se((InHR))			
Daniela Cimino 2008	Italy	BC	127	High	67			1.36	0.74–2.50	0.31	0.31					qRT-PCR	≥the median value	7
				Low	60													
XiaoMin Wang 2013	China	EC	133	High				1.28	0.77–2.14	0.25	0.26					IHC	≥weak expression	7
				Low														
S. Soysal 2013	Switzerland	BRC	1402	High				0.78	0.65–0.93	−0.251	0.091					IHC	≥5%	9
				Low														
Queting Chen 2014	China	CC	96	High	62	34	28	3.15	1.03–9.61	1.146	0.57					IHC	Overall scores ≥ 3	7
				Low	34	27	7											
Na Wang 2015	China	GC	131	High	68	22	46	1.72	1.03–2.86	0.54	0.261					qRT-PCR	a copy number ≥ 4	9
				Low	63	29	34											
Hongbing Liu 2015	China	NSCLC	63	High	32	15	17	2.05	1.02–4.12	0.718	0.356					IHC	Overall scores ≥ 2	9
				Low	31	24	7											
Shichong Liao 2016	China	BRC	67	High	49	28	21									Western blot		7
				Low	18	14	4											
Xue Liu 2016	China	BRC	128	High	58	46	12					0.86	0.32–2.34	−0.15	0.51	IHC	≥4	9
				Low	70	53	17											
Elmer Hoekstra 2016	Netherlands	CC	372	High	140	61	79	1.29	0.99–1.68	0.252	0.135	1.36	1.05–1.75	0.304	0.131	IHC	score > 6	9
				Low	232	145	87											
HaoWei Teng 2016	China	CC	242	High	141			1.58	1.11–2.25	0.46	0.18	2.69	0.62–11.7	0.99	0.75	IHC		7
				Low	101													
CarolineE 2019	Spain	NB	44	High	14	5	9	1.16	0.31–4.32	0.15	0.67					IHC	staining positive cells	7
				Low	27	21	6											
Tao Jin 2019	China	GM	311					1.69	1.04–2.73	0.522	0.246					qRT-PCR	a copy number ≥ 4	9
Qi Xu 2019	China	PC	118	High	67	52	15	1.35	0.84–2.16	0.30	0.24					IHC	Overall scores ≥ 5	7
				Low	51	49	2											
Yichuan Chen 2020	China	NSCLC	84	High				0.66	0.52–0.85	−0.408	0.125					IHC		7
				Low														
Jing Chen 2020	China	GC	347	High	86			1.48	0.84–2.59	0.389	0.287					qRT-PCR		9
				Low	261													
		EC	115	High	27			1.12	0.77–1.62	0.112	0.189					qRT-PCR		9
				Low	88													
		CC	273	High	74			0.72	0.37–1.43	−0.323	0.346					qRT-PCR		9
				Low	199													
Ben Davidson 2020	Norway	SC	62	High								0.73	0.50–1.05	−0.32	0.19	IHC	score > 4	7
				Low														
		MMa	29	High				1.67	0.69–3.99	0.51	0.446							7
				Low														
Qiang Wang 2021	China	MM	44	High	23			1.68	0.61–4.66	0.52	0.52					qRT-PCR		7
				Low	21													

BC = bladder cancer, BRC = breast cancer, CC = colorectal carcinoma, CI = confidence interval, DFS = disease free survival, EC = esophagus carcinoma, GC = gastric carcinoma, GM = glioma, HR = hazard ratio, IHC = immunohistochemistry, MM = malignant melanoma, MMa = malignant mesothelioma, NB = neuroblastoma, NSCLC = non-small cell lung carcinoma, OS = overall survival, PC = pancreatic carcinoma, PTP1B = protein tyrosine phosphatase 1B, SC = serous carcinoma, TNM = TNM classification.

### 3.2. Meta-analysis

#### 3.1.1. Association between PTP1B expression and clinical stages of solid tumors.

**Figure 2.**
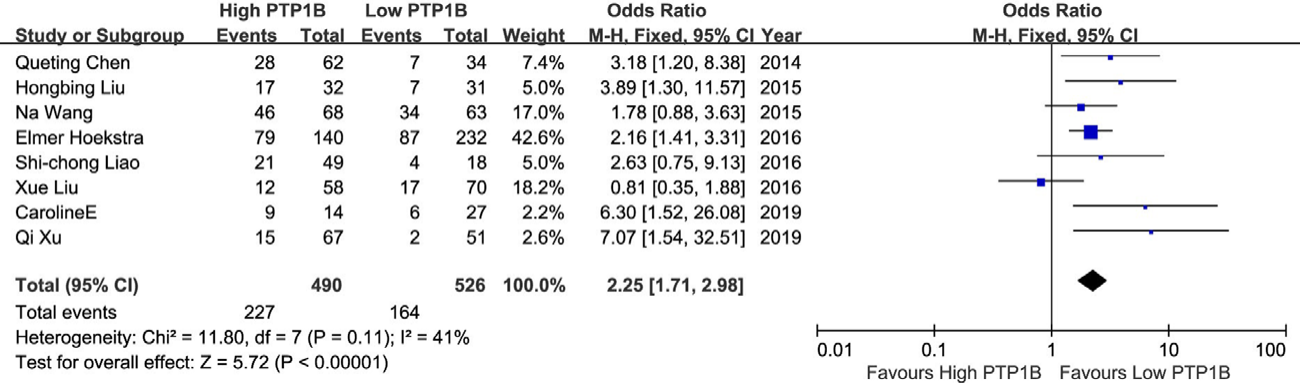
A forest plot to assess the effect of PTP1B on clinical stages of solid tumors. PTP1B = protein tyrosine phosphatase 1B.

The correlation between PTP1B expression and clinical stages was reported in 8 studies. A fixed effect model was adopted because there was no obvious heterogeneity (*P* = .11, *I*^2^ = 41%). The combined OR showed that higher PTP1B expression was significantly associated with later clinical stages of solid tumors (OR 2.25, 95%CI: 1.71–2.98, *P* ＜ .001) (Fig. 2). Publication bias was used to evaluate the reliability of the results. The symmetrical funnel plot revealed that the pooled clinical stage did not show publication bias. Egger test (*t* = 1.93, *P* = .102) also indicated no publication bias (Table 2).

**Table 2:** The publication bias test including literatures.

	Coef	95% CI	*t*	*P* value
TNM	1.208	−0.324 to 2.739	1.93	.102
DFS	−0.978	−8.625 to 8.429	−0.05	.965
OS	2.028	0.701 to 3.354	3.28	.005

DFS = disease free survival, OS = overall survival, TNM = TNM classification.

#### 3.1.2. PTP1B expression and DFS of solid tumors.

**Figure 3.**
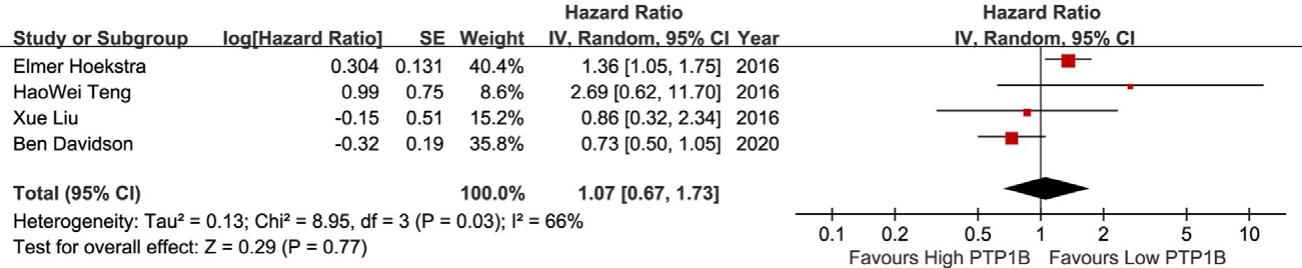
A forest plot to assess the effect of PTP1B on DFS of solid tumors. DFS = disease free survival, PTP1B = protein tyrosine phosphatase 1B.

The relationship between PTP1B and DFS of solid tumors was estimated in 4 studies. The random effect model was used due to the obvious heterogeneity (*P* = .03, *I*^2^ = 66%). The HR for DFS detrimental with high PTP1B expression compared with low PTP1B expression was 1.07 (95%CI: 0.67–1.73) for solid tumors (Fig. 3). For the pooled results with heterogeneity, the stability of the results was determined by sensitivity analysis. When sensitivity analysis was performed, the combined HR of PFS changed significantly (Table 3), indicating that this result should be treated with caution. The symmetrical funnel plot revealed that no publication bias was found in combined PFS. Egger test (*t* = 1.93, *P* = .102; *t* = −0.05, *P* = .965) also indicated no publication bias (Table 2).

**Table 3:** Sensitivity analysis for PFS and OS.

Outcome	Study omitted	Resulting HR (95% CI)	heterogeneity
PFS	Ben Davidson2020	1.34 (1.05–1.72)	(*P* = .44, *I*^2^ = 0%)
	Elmer Hoekstra2016	0.89 (0.51–1.56)	(*P* = .24, *I*^2^ = 31%)
	HaoWei Teng2016	0.98 (0.60–1.62)	(*P* = .02, *I*^2^ = 73%)
	Xue Liu2016	1.13 (0.64–2.00)	(*P* = .01, *I*^2^ = 77%)
OS	Daniela Cimino2008	1.33 (1.08–1.63)	(*P* ＜ .001, *I*^2^ = 62%)
	XiaoMin Wang2013	1.34 (1.09–1.65)	(*P* ＜ .001, *I*^2^ = 62%)
	S. Soysal2013	1.39 (1.22–1.58)	(*P* = .70, *I*^2^ = 0%)
	Queting Chen2014	1.30 (1.07–1.57)	(*P* = .002, *I*^2^ = 59%)
	Hongbing Liu2015	1.30 (1.07–1.58)	(*P* = .002, *I*^2^ = 60%)
	Na Wang2015	1.30 (1.07–1.59)	(*P* = .002, *I*^2^ = 60%)
	HaoWei Teng2016	1.31 (1.07–1.60)	(*P* = .002, *I*^2^ = 59%)
	Elmer Hoekstra2016	1.34 (1.08–1.67)	(*P* ＜ .001, *I*^2^ = 62%)
	CarolineE2019	1.34 (1.09–1.63)	(*P* ＜ .001, *I*^2^ = 63%)
	Tao Jin2019	1.31 (1.07–1.60)	(*P* = .002, *I*^2^ = 60%)
	Qi Xu2019	1.33 (1.08–1.64)	(*P* ＜ .001, *I*^2^ = 62%)
	Jing Chen2020	1.32 (1.08–1.62)	(*P* ＜ .001, *I*^2^ = 62%)
	Ben Davidson2020	1.32 (1.08–1.61)	(*P* ＜ .001, *I*^2^ = 62%)
	Jing Chen2020	1.36 (1.10–1.68)	(*P* ＜ .001, *I*^2^ = 63%)
	Jing Chen2020	1.37 (1.12–1.68)	(*P* ＜ .001, *I*^2^ = 61%)
	Qiang Wang2021	1.32 (1.08–1.61)	(*P* ＜ .001, *I*^2^ = 62%)

CI = confidence interval, IHC = immunohistochemistry, HR = hazard ratio, OS = overall survival, PFS = progression free survival.

#### 3.1.3. PTP1B expression and OS of solid tumors.

**Figure 4.**
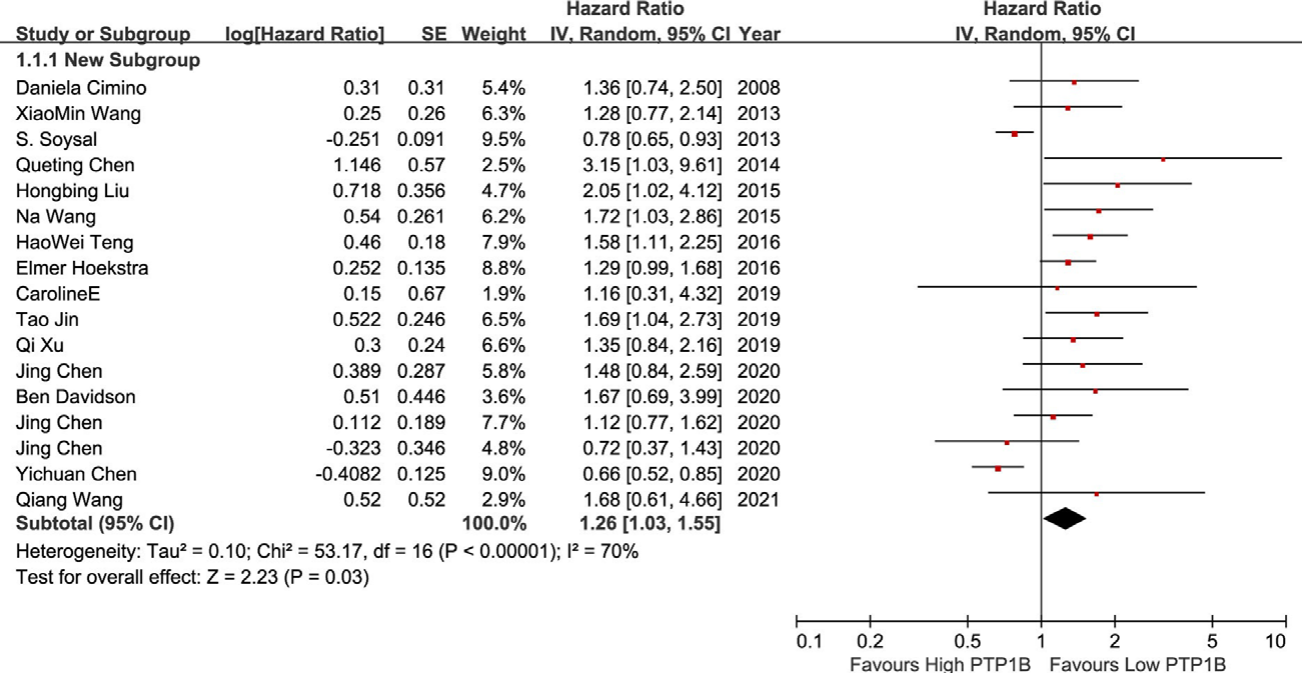
A forest plot to assess the effect of PTP1B on OS of solid tumors. OS = overall survival, PTP1B = protein tyrosine phosphatase 1B.

**Figure 5.**
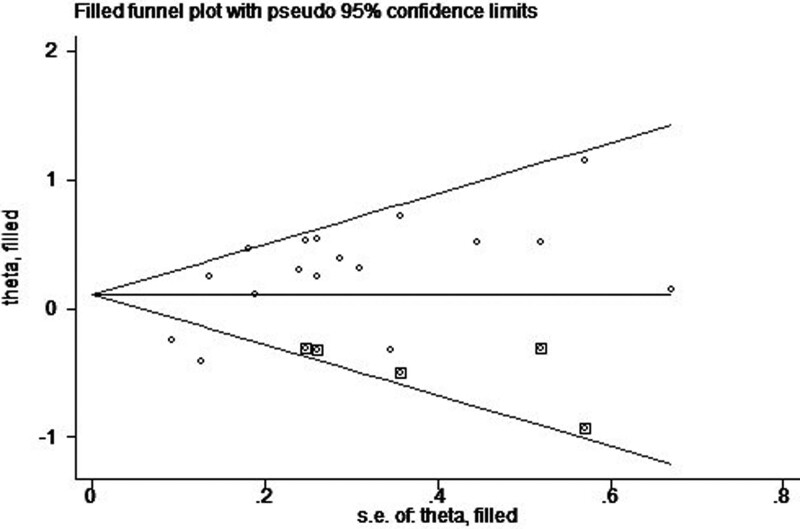
Filled funnel graph for the assessment of publication bias of OS. OS = overall survival.

We pooled data from 8 studies, using a random effect model with heterogeneity between studies (*P* = .01, *I*^2^ = 60%). The HR of OS of solid tumors with high PTP1B expression versus low PTP1B expression was 1.26 (95%CI: 1.03–1.55, *P* = .03) (Fig. 4). Similarly, when a single study was excluded in turn, sensitivity analysis revealed that the pooled HR of OS did not change significantly (Table 3). The funnel plot demonstrated that there was significant publication bias for OS (*t* = 3.28, *P* = .005). So, the trim and fill method were carried out to recalculate our pooled HR. The analysis suggested that there was some evidence of asymmetry (5 studies trimmed), and the overall effect was strongly influenced by 5 studies (Fig. 5). The adjusted HR was 1.116 (95% CI: 0.930–1.339), which was inconsistent with our original risk estimate, indicating that our results should be interpreted cautiously.

### 3.3. Subgroup analysis

**Figure 6.**
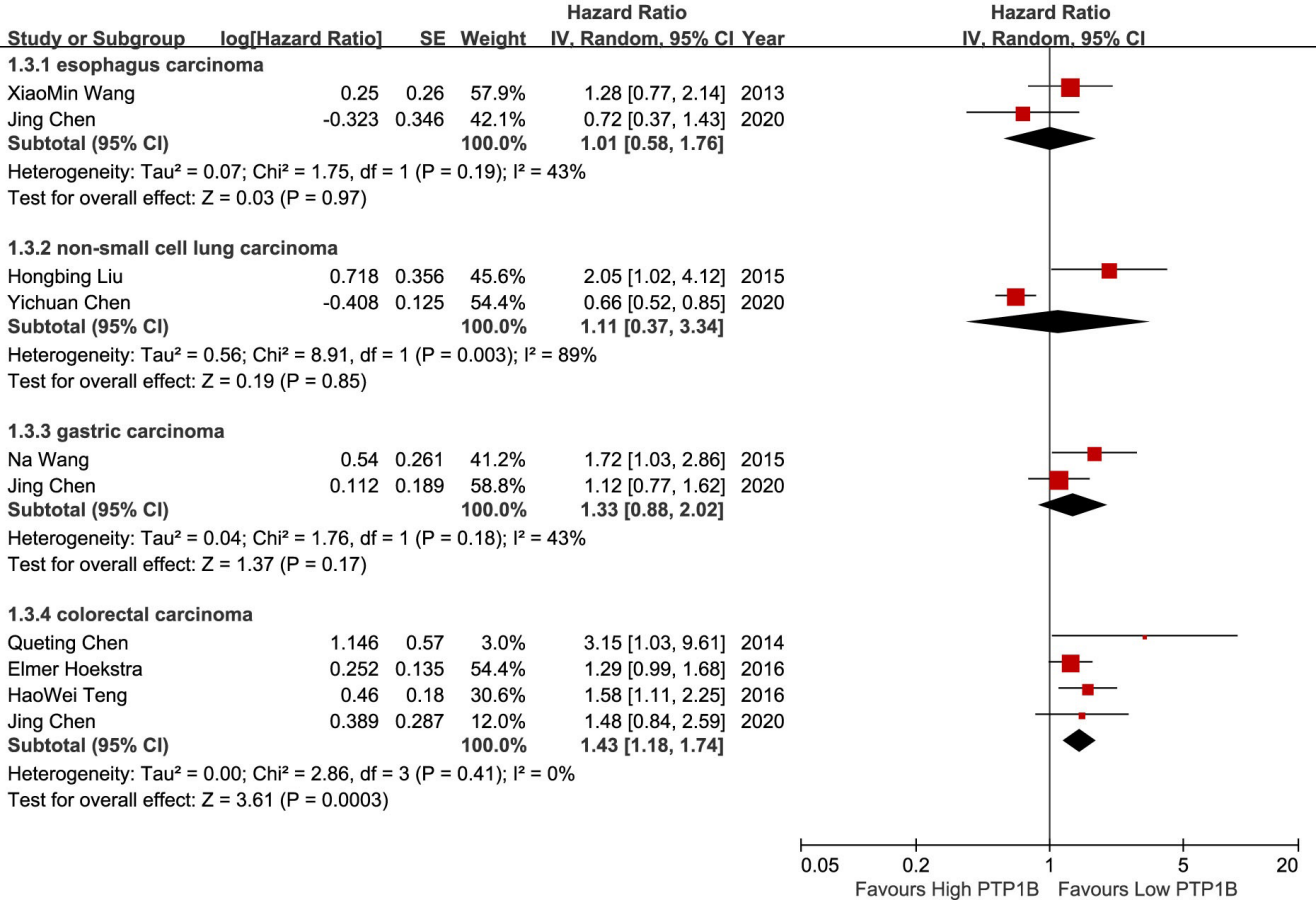
Forrest plots to assess the effect of PTP1B on OS in different tumors of subgroups. OS = overall survival, PTP1B = protein tyrosine phosphatase 1B.

Considering the large heterogeneity when merging OS, subgroup analysis was performed. The heterogeneity decreased significantly because these studies grouped by tumor types. In subgroup analysis, high PTP1B expression did not correlated with OS in esophagus carcinoma (HR = 1.01; 95%CI: 0.58–1.76; *P* = .97; *I*^2^ = 43%, *P* = .19), non-small cell lung carcinoma (HR = 1.11; 95%CI: 0.37–3.34; *P* = .85; *I*^2^ = 89%, *P* = .003) and gastric carcinoma (HR = 1.33; 95%CI: 0.88–2.02; *P* = .17; *I*^2^ = 43%, *P* = .18). High PTP1B expression was remarkably associated with poor OS in colorectal carcinoma (HR = 1.43; 95%CI: 1.18–1.74; *P* = .003; *I*^2^ = 0%, *P* = .41) (Fig. 6).

## 4. Discussion

PTP1B is initially purified from human placenta with a 37 kDa catalytic domain. Its C-extended-end is composed of 2 areas. One is a proline rich region, interacting with Src homologous 3 domains to recruit substrates, the other is the hydrophobic region, inserted into the endoplasmic reticulum membrane to anchor PTP1B on the cytoplasmic side of the endoplasmic reticulum.^[7]^ Some reports founded that the PTP1B expression was associated with certain clinical characteristics, such as clinical stages, and the prognosis of solid tumors including DFS/PFS and OS. However, the results were controversial. So, this study discussed the role of PTP1B in the prognosis of solid tumor patients, avoiding possible bias.

This meta-analysis supported that higher PTP1B expression was significantly associated with later clinical stage of solid tumors. This study could not draw a conclusion that PTP1B was, or not associated with DFS and OS of solid tumors because of heterogeneity and publication bias. Subgroup analysis revealed that high PTP1B expression did not correlated with OS in esophagus carcinoma, non-small cell lung carcinoma and gastric carcinoma. High PTP1B expression was remarkably associated with poor OS in colorectal carcinoma. All these suggested that PTP1B might play a role in solid tumors by various mechanisms.

The functions of PTPN genes primarily depend on peptidyl-tyrosine dephosphorylation and protein tyrosine phosphatase activity.^[13]^ In neuroblastoma, the knock-down of PTPN1 affects the tyrosine phosphorylation and neuroblastoma cells proliferation.^[21]^ PTP1B induces down-regulation of paired like homeodomain expression by acting on phospho(Y)-paired like homeodomain in colorectal carcinoma.^[22]^ PTP1B promotes the proliferation and metastasis of cells via inducing src and extracellular regulated protein kinases activation in non-small cell lung cancer.^[23]^ In contrast, it was reported that PTP1B degraded by CAPN1 (calpain) promoted malignant behavior and erlotinib resistance of lung adenocarcinoma through phosphorylating cellular-mesenchymal epithelial transition factor and phosphoinositide-3-kinase regulatory subunit 2.^[24]^ PTP1B accelerates cell growth and invasiveness through the activation of MAPK (mitogen-activated protein kinase) and phosphatidylinositide 3-kinases/ protein kinase B signaling pathways in gastric cancer^[25]^ and glioma.^[26]^ PTP1B is also regulated by miR-146b, controlling the proliferation and apoptosis in gastric cancer.^[27]^ Inhibiting PTP1B through targeting the PKM2/AMPK/mTOC1 (pyruvate kinase isozymes M2/Adenosine 5’-monophosphate-activated protein kinase/mammalian target of rapamycin complex 1) pathway restrains pancreatic cancer progression.^[28]^ PTP1B interacting with Src promote the metastasis of cells in melanoma.^[29]^ PTP1B up-regulates the dephosphorylated level of signal transducer and activator of transcription and the expression of regulated upon activation normal T cell expressed and secreted factor and accelerates cells migration and invasion,^[30]^ also inhibits phosphatase and tensin homolog deleted on chromosome 10 and up-regulates matrix metalloproteinases^[31]^ in breast cancer. PTP1B promotes tumor survival under hypoxia condition through regulating RNF213 (ring finger protein) to control non-mitochondrial oxygen consumption.^[32]^ Calreticulin regulates PTP1B transcription through Stat5a to induce metastatic phenotypesin in esophagus carcinoma cells.^[33]^ These different mechanisms might produce different prognostic effects of PTPN on solid tumors.

This study had several limitations. First, there were substantial differences among the included studies, leading to great heterogeneity. Therefore, the estimated results should be treated with caution, even if hierarchical analysis was carried out. Second, the population included in the studies was mainly from China, which could not commendably represent the global population. At last, publication bias for OS was a concern.

In conclusion, higher PTP1B expression was associated with later clinical stages of solid tumors. Subgroup analysis revealed that high PTP1B expression was remarkably associated with poor OS in colorectal carcinoma, only. This study could not draw a conclusion that PTP1B was, or not associated with DFS and OS of solid tumors because of heterogeneity and publication bias. Whether PTP1B can be used as a biomarker for predicting the prognosis of solid tumors needs further study.

## Author contributions

Jiupeng Zhou and Quanli Dou made contributions to conception and design, publication search, quality evaluation, data collection, statistics and manuscript writing. Yongfeng Zhang and Heng Liu made contributions to statistics and editors, and Hui Guo contributed to conception, design, statistics and editing.

**Conceptualization:** Jiupeng Zhou, Hui Guo, Quanli Dou.

**Data curation:** Yongfeng Zhang, Heng Liu.

**Formal analysis:** Heng Liu.

**Investigation:** Quanli Dou.

**Methodology:** Jiupeng Zhou, Hui Guo, Yongfeng Zhang, Heng Liu.

**Resources:** Jiupeng Zhou, Yongfeng Zhang, Heng Liu.

**Supervision:** Jiupeng Zhou.

**Validation:** Jiupeng Zhou.

**Writing – original draft:** Jiupeng Zhou.

**Writing – review & editing:** Jiupeng Zhou, Hui Guo, Quanli Dou.
